# The Reliability and Reproducibility of a New Revised Edelson Classification

**DOI:** 10.1111/os.13375

**Published:** 2022-08-04

**Authors:** Jialiang Guo, Weichong Dong, Yali Zhou, Meishuang Shang, Sifan Yang, Xiaojuan Zhang, Zhiyong Hou, Yingze Zhang

**Affiliations:** ^1^ The School of Medicine Nankai University Tianjin PR China; ^2^ Department of Orthopaedics The Third Hospital of Hebei Medical University Shijiazhuang PR China; ^3^ Department of Pharmacy The Second Hospital of Hebei Medical University Shijiazhuang PR China; ^4^ Faculty of Medicine and Health Chinese Academy of Engineering Beijing PR China; ^5^ NHC Key Laboratory of Intelligent Orthopeadic Equipment The Third Hospital of Hebei Medical University Shijiazhuang PR China

**Keywords:** Bicipital groove fracture, CT reconstruction, Edelson classification, Revised Edelson classification

## Abstract

**Objective:**

Edelson classification is a 3D classification of proximal humeral fractures, but there is a scarcity of application of this classification in large samples, and the accuracy of classification was also not testified. The objective of this research was to verify whether a revised Edelson classification produces satisfactory agreement for proximal humeral fracture classification in adult patients.

**Methods:**

A total of 827 proximal humeral fractures (304 male and 520 female patients, 58.0 ± 16.2 years) were found retrospectively from January 2014 to December 2019, and classified according to the traditional and newly proposed Edelson classification. The three‐dimensional CT images were processed, rotated and visualized within software. Five shoulder surgeons classified each fracture. After data collection, radiographic classifications results were compared by inter‐ and intraobserver analysis with the method of weighted kappa coefficients. Fracture classification based on Edelson and revised Edelson classification was presented and compared.

**Results:**

The mean *k* value for the interobserver reliability was 0.748 (range, 0.583 to 0.958) compared with Edelson classification (0.548, range, 0.48 to 0.635), indicating satisfactory agreement. The mean *k* value for intraobserver reliability was 0.906 (range, 0.823 to 0.943) compared with Edelson classification (0.762, range, 0.666 to 0.808), indicating excellent agreement when using the newly revised Edelson classification. The mechanism was categorized as the shoulder being in a position of forward flexion, abduction, and internal rotation in Edelson I‐IV and bicipital fractures. For the greater tuberosity fracture, the mechanism was classified into two mechanisms based on the presence of a combined dislocation. Bicipital groove fracture is a commonly observed fracture pattern, and included in the revised Edelson classification.

**Conclusions:**

The revised Edelson classification proposed was more in line with the injury mechanism of the fracture, was beneficial in identifying more fracture types such as bicipital groove fracture, and verified to be a good proximal humeral fracture classification with good reliability compared with the traditional Edelson classification.

## Graphical abstract

A total of 1907 consecutive adult proximal humeral fracture patients with available plain X‐rays and CT scans were identified and included in our research. At last, 824 patients (827 fractures) with complete data were included in the sample. The proximal humeral fracture was classified according to the Edelson classification, try to find the deficiency in the traditional Edelson classification, and then propose a new revised classification which improve the rationality and reliability of the classification. And try to explain the rationality of the new classification.

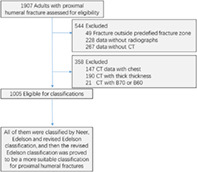

## Introduction

Proximal humeral fracture (PHF) is a common fracture that has a great effect on patients' health and quality of life; it accounts for approximately 4% to 5% of all fractures, and 33% of it occurs in the elderly.^1^, ^2^ Numerous classification systems, including the Neer and AO classifications, have been proposed to guide the treatment of proximal humeral fractures. Among them, Neer's classification was the most commonly accepted classification given its simplicity.^3^, ^4^ However, accurate measurements of displaced or angulated fractures make this system difficult to apply in the clinic. Sumrein *et al*. reported that surgeons could identify 2‐part surgical neck fractures from multi‐fragmented fractures based on the Neer classification.^5^, ^6^ However, there is a growing consensus that the Neer classification does not produce good inter‐ and intraobserver reliability and has limited guidance in the establishment of treatment strategy.^7^, ^8^ The arbitrary definition of “displacement,” results to a negative effect and amplified in complex fracture (3‐ and 4‐part fractures) of which all fractured parts could be identified as displaced according to the NC definition.^8^

It was difficult to understand the 3D PHF on previous classifications methods which based on two‐dimensional images (Neer classification). Small or delicate changes in rotation and positioning can result in considerable disagreement in the interpretation of standard radiographs. Therefore, a classification system that has better reliability and reproducibility for fractures is warranted and helpful for orthopedic surgeons in patient selection. Furthermore, CT (computed tomography) and 3D CT construction images with high resolution provide a much clearer view of the pattern of proximal humeral fractures. Edelson proposed a new three‐dimensional classification for fractures of the proximal humerus. The results concluded that it was useful in classifying these injuries with reasonable inter‐ and intraobserver reliability.^9^

It could be hypothesized that patients with PHF would benefit from this 3D classification, but there is a scarcity of application of the Edelson classification in large samples, and the accuracy of classification was also not testified. Furthermore, it was found that there are still some essential fracture types cannot be classified based on the Edelson classification. The objective of this research was to: (i) test whether this Edelson classification produces satisfactory agreement for proximal humeral fracture; (ii) try to develop a new revised Edelson classification system if it did not conclude all main fracture types; and (iii) to testify the rationality of the newly revised Edelson classifications.

## Materials and Methods

### Study Design and Eligibility Criteria

In this single‐center retrospective study, the anonymous data of 1907 patients with unique identifying numbers who sustained PHF were obtained and evaluated from January 2014 to December 2019. The research was conducted in one orthopedics center in March 2021. A total of 1907 consecutive adult proximal humeral fracture patients with available plain X‐rays and CT scans were identified and included in our research. All of these anonymous data were collected and assessed by the authors over a period of 8 months. Primary approval was received from the Regional Ethics Committee of our hospital (W2021‐069‐1), and the study was conducted in accordance with the Declaration of Helsinki. Informed verbal consent was obtained through telephone calls from all patients enrolled in the study (Clinical trial number NCT04523415).

Inclusion criteria for the study were as follows: (i) clear record of demographic data; (ii) initial X‐ray, and CT imaging data were complete; (iii) three‐dimensional construction with mimics (with CT of B30); and (iv) patients older than 18 years with displaced PHF occurring less than 3 weeks before allocation and treatment.

The exclusion criteria for the study were as follows: (i) patients with the presence of neurological disease (syringomyelia); (ii) pathological fracture (other than osteoporosis); and (iii) not a resident in the hospital catchment area.

### Interventions

Following study enrollment, 5 trained researchers helped to obtain patient demographic information, such as age, gender, and injury mechanism. CT examinations of patients were scanned in a multislice spiral computed tomography scanner and then collected, and the exact technique of CT acquisition was the same at our hospital. The slice thickness was set as 1–2 mm and reconstructed in orthogonal planes. Initial varus displacement was identified as <130°, and initial valgus displacement was >130°, and the neck shaft angle of 130° was identified as normal.^10^ The classification of proximal humeral fractures was determined by CT. Furthermore, two surgeons helped us to confirm the classification on the basis of standard CT. Three‐dimensional CT images were processed, rotated and visualized with Radiant software (Medixant, Poznan, Poland) as reported by Edelson *et al*.^9^ In this format, simultaneous 4‐quadrant views of the fracture are displayed so that the injury is observed from the front, side, back and from above in one composite picture. The 3D images reconstructed can be rotated freely to measure the length, width and depth on desired planes.

### Data Collection

To describe and verify the fracture population in this research, five independent and blinded shoulder surgeon specialists characterized all study fractures from CT and 3D reconstructions using the Edelson classification.^9^ Edelson I was described as a two‐part fracture that involves the shaft and proximal humerus. Edelson II included fractures located at the surgical neck and greater tuberosity. Edelson III described shield fractures. For IV, it means shield fracture variants. Edelson V was identified as isolated greater tuberosity (GT) fractures.

The revised Edelson classification was identified as: Edelson I′ (same as Edelson I), Edelson II′ (same as Edelson II), Edelson III′ (shield and shield variant fracture), Edelson IV′ (bicipital fracture), and Edelson V′ (tuberosity fracture).

### Fracture Classification

All radiographs and computed tomographies were classified by the same five shoulder surgeons in the upper limb. The identification numbers of all patients were removed before classification to make sure the independence of each typing process. Radiographies were first sent digitally to each surgeon, approximately 3 weeks after tomographies. Each surgeon classified each fracture using Neer and traditional Edelson classification, and classified it in tables.

### Outcome Measures

The characteristics such as patients' distribution of age and sex, and injury mechanism of each fracture type in the Edelson classification was collected and compared. After data collection, radiographic and tomographic classifications were compared by inter‐ and intraobserver analysis with different classification (Neer, Edelson and revised Edelson classifications).

### Statistical Analysis

The number of the chosen category for each fracture together with assessment of dislocation was recorded on separate forms. The inter‐ and intraobserver reliability was statistically analyzed by weighted kappa coefficients (SPSS 21, IBM, Armonk, NY, USA). The coefficient values were classified as follows: 0–0.19 (unsatisfactory), 0.20–0.39 (low agreement), 0.40–0.59 (moderate agreement), 0.60–0.79 (satisfactory agreement) and 0.80–1.00 (perfect).

## Results

### Interobserver and Intraobserver Reliability

**Fig. 1:**
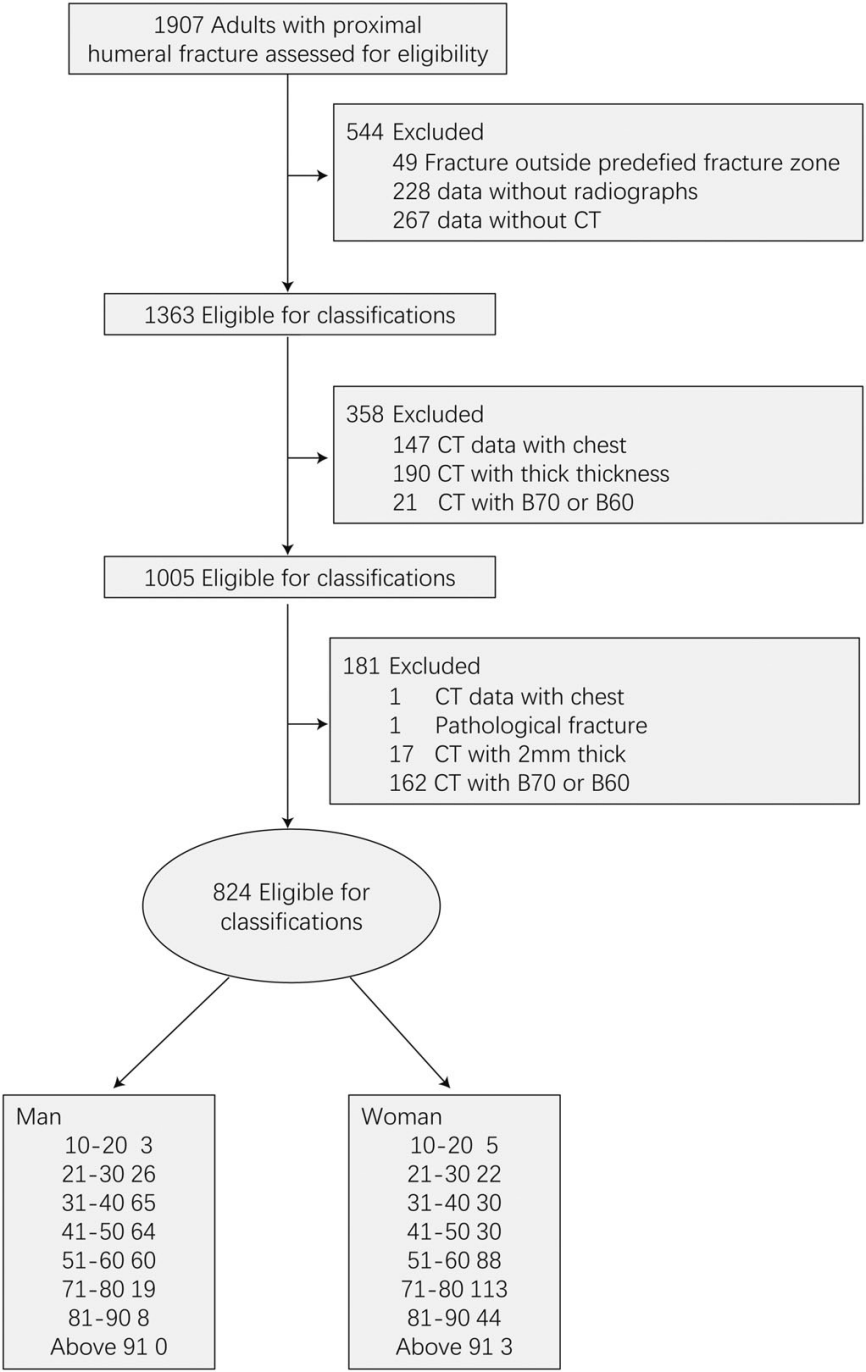
Enrollment and analysis of patients in the retrospective research

Of the 1907 patients screened for inclusion, 824 patients (827 fractures) with complete data were included in the sample (Fig. 1). The CT of the 827 fractures was evaluated by five shoulder surgeons. The mean *k* value for the interobserver reliability of the revised Edelson classification was 0.748 (range, 0.583 to 0.958), indicating satisfactory agreement. The mean *k* value for the interobserver reliability of the Edelson classification was 0.548 (range, 0.48 to 0.635), indicating moderate agreement. The mean *k* value for the interobserver reliability of the Neer classification was 0.332 (range, 0.281 to 0.462), indicating low agreement.

Regarding intraobserver evaluations, the mean *k* value of Neer classification was 0.437, indicating moderate agreement (range, 0.392 to 0.531). There was substantial agreement for the intraobserver reliability of the Edelson classification (0.762, range, 0.666 to 0.808). However, the mean *k* value was 0.906 (range, 0.823 to 0.943), indicating excellent agreement when using the revised Edelson classification.

### Classification Results

During the study period, 824 patients or 827 proximal fractures met the inclusion criteria. There were 304 male and 520 female patients, and the mean age was 58.0 ± 16.2 years (range, 18 to 93 years). There was a high proportion of complex fracture configurations with 88.4% including a tuberosity fracture (731 fractures) and 11.4% involving joint dislocation. Among patients who were allocated to Edelson classification, 95 fractures were categorized as Edelson I, 294 fracture patients were classified as Edelson II, and only 14 fractures were classified as Edelson III. In total, 99 fractures were identified as Edelson IV. Furthermore, 176 fractures were classified as Edelson V. However, 141 other fractures could not be classified according to traditional Edelson classification, but 119 of them can be classified as bicipital groove fractures by the new revised Edelson classification.

### Two‐part Fractures (Edelson I)

**Fig. 2:**
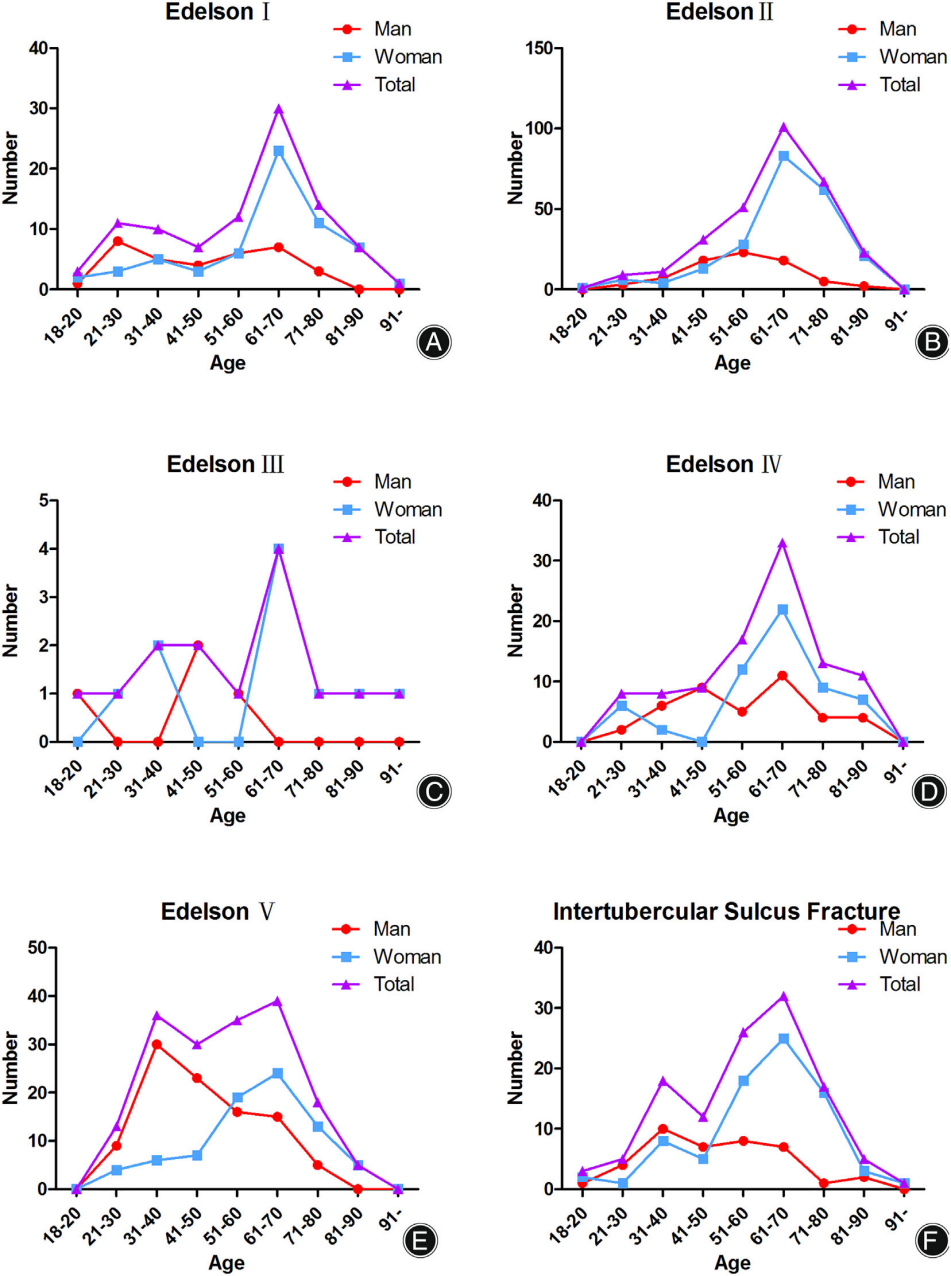
The distribution of fracture patients with different ages in each classification. (A) The number of fractures in each age group of Edelson I. (B) The number of fractures in each age group of Edelson II patients. (C) The number of fractures in each age group of Edelson III patients. (D) The number of fractures in each age group of Edelson IV patients. (E) The number of fractures in each age group of Edelson V patients. (F) The number of fractures in each age group of intertubercular sulcus fractures

**Fig. 3:**
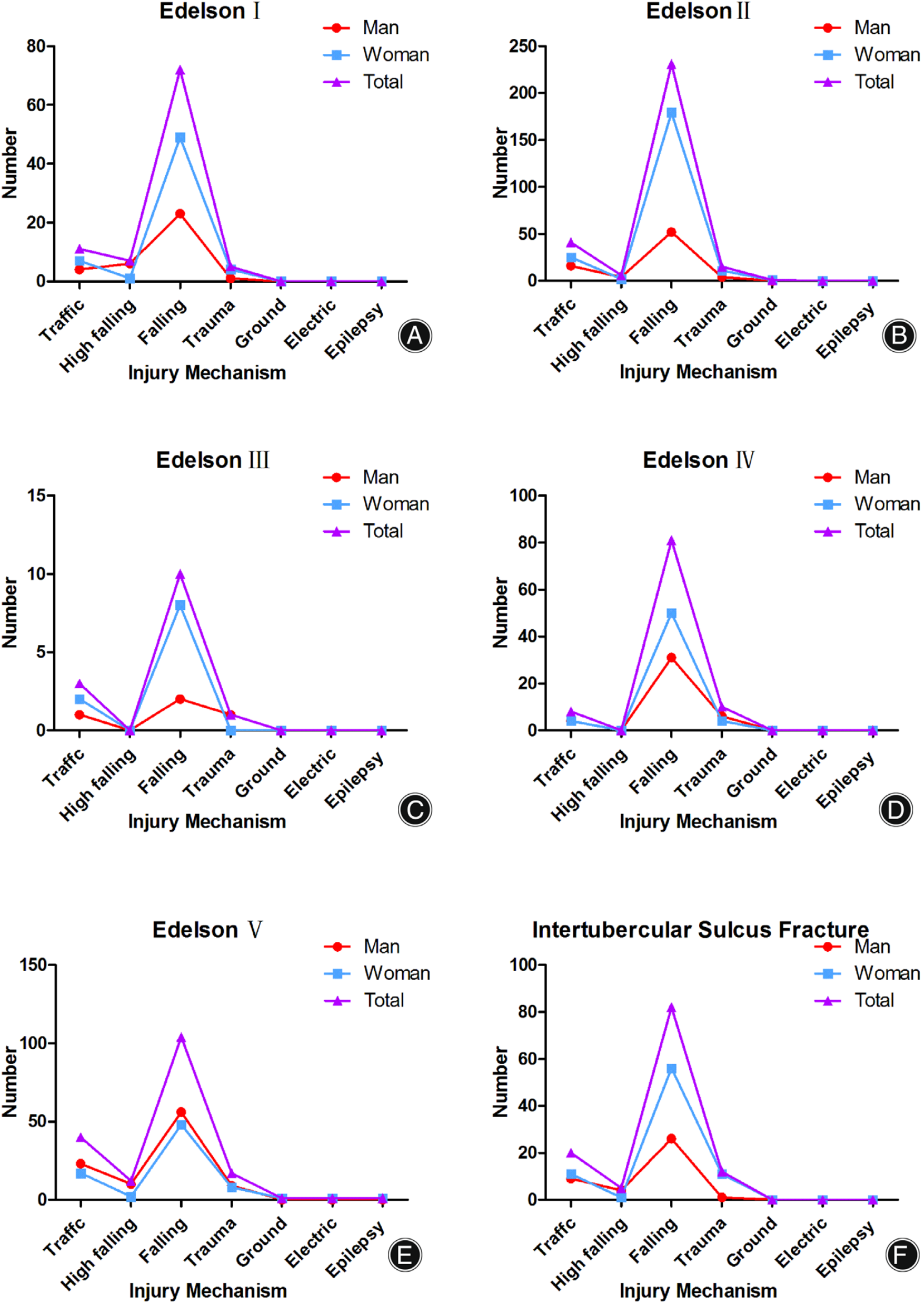
The distribution of fracture patients with different injury mechanisms in each classification. (A) The number of fractures in each injury mechanism of Edelson I patients. (B) The number of fractures in each injury mechanism of Edelson II patients. (C) The number of fractures in each injury mechanism of Edelson III patients. (D) The number of fractures in each injury mechanism of Edelson IV patients. (E) The number of fractures in each injury mechanism of Edelson V patients. F, The number of fractures in each injury mechanism of intertubercular sulcus fracture

There were 95 fractures (11.48%) classified as Edelson I. The highest risk age group of women was 61–70 years group, and the highest risk age group of men was 21–30 years group (8 patients) (Fig. 2(A)). Injuries occurred most commonly by falling injury (Fig. 3(A)).

### Three‐part Fractures (Edelson II)

Among 294 fractures, 117 fractures were located in the valgus position, and 124 fractures were located in the neutral position. Furthermore, 30 fractures were located in the varus position, 21 fractures were injured with anterior dislocation, and two fractures were subluxed (Table 1). The highest risk age group and that of women was 61–70 years group, and the highest risk age group of men was 51–60 years group (23 patients) (Fig. 2(B)). Injuries occurred most commonly by falling injury (Fig. 3(B)).

**TABLE 1:** The position of fracture in each main classification in Edelson classification

	Valgus	Neutral	Varus	Dislocation (subluxation)
Edelson I	37	32	24	2
Edelson II	117	124	30	23
Edelson III	3	6	3	2
Edelson IV	31	39	6	23
Bicipital groove	38	53	14	14
Total	226	254	77	64

### Shield Fracture (Edelson III)

Only 14 fractures were identified as shield fractures. Three fractures were located in the valgus position, and six fractures were located in the neutral position. Furthermore, three fractures were located in the varus position, and two fractures were injured with anterior dislocation (Table 1). The highest risk age group and that of women was 61–70 years group, and the highest risk age group of men was 41–50 years group (23 patients) (Fig. 2(C)). Injuries occurred most commonly by falling injury (Fig. 3(C)).

### Shield Variant Fracture (Edelson IV)

The highest risk age group of women was 61–70 years old (Fig. 2(D)). Injuries occurred most commonly by falling injury (Fig. 3(D)). Only six fractures were identified as Edelson IV‐1, which was named a four‐part shield fracture. Seventy‐three fractures were classified as Edelson IV‐2, which was identified as shattered shield fracture. Furthermore, only 20 fractures were classified as Edelson IV‐3, and these fractures were termed as head spilt fractures.

### Isolated Fractures of the Greater Tuberosity (Edelson V)

Among those GT fractures (Edelson V), the highest risk age group of women was 61–70 years old, and the highest risk age group in men was 31–40 years old (30 patients) (Fig. 2(E)). Injuries occurred most commonly *via* fall injury (Fig. 3(E)). For those GT fractures with dislocations, 28 patients had anterior fracture dislocations, two had subluxation, and one patient had posterior fracture dislocations.

### Proximal Bicipital Groove Fractures (Intertubercular Sulcus Fracture)

In total, 119 fractures were identified as bicipital groove fractures. The highest risk age group of women were 61–70 years old, and the highest risk age group of men was 31–40 years old (10 patients) (Fig. 2(F)). Injuries occurred most commonly by falling injury (Fig. 3(F)).

### Other infrequent types of fractures

Three fractures were observed with only shield fracture shapes. The greater tuberosity split along with the bicipital groove and the lesser tuberosity, but the surgical neck was intact. Therefore, it was called A shell fracture. Fifteen fractures were found with greater and lesser tuberosity fractures. Furthermore, 15 fractures combined with lesser tuberosity were also observed. Only one independent lesser tuberosity fracture was found. Eight fractures were identified as humeral shaft spiral fractures combined with greater tuberosity fractures.

## Discussion

The research revealed that there were a good inter‐ and intra‐agreement about the revised Edelson classification compared with the traditional ones, and it was also found that the traditional classification did not contain proximal bicipital groove fracture, which was commonly found in our research. Furthermore, it was concluded that the main injury mechanism of Edelson I to IV was the posture of the parachute reflex. The GT fractures was mainly caused by impingement of the greater tuberosity against the acromion. For dislocated GT fractures, shearing against the glenoid rim was considered as the main injury mechanism. The research changed the traditional comprehension about proximal fracture, and made morphology of the fracture more easily being understood.

### The Traditional Edelson Classification System Does Not include Bicipital Groove Fracture, So a New Revised Edelson Classification was Proposed

**Fig. 4:**
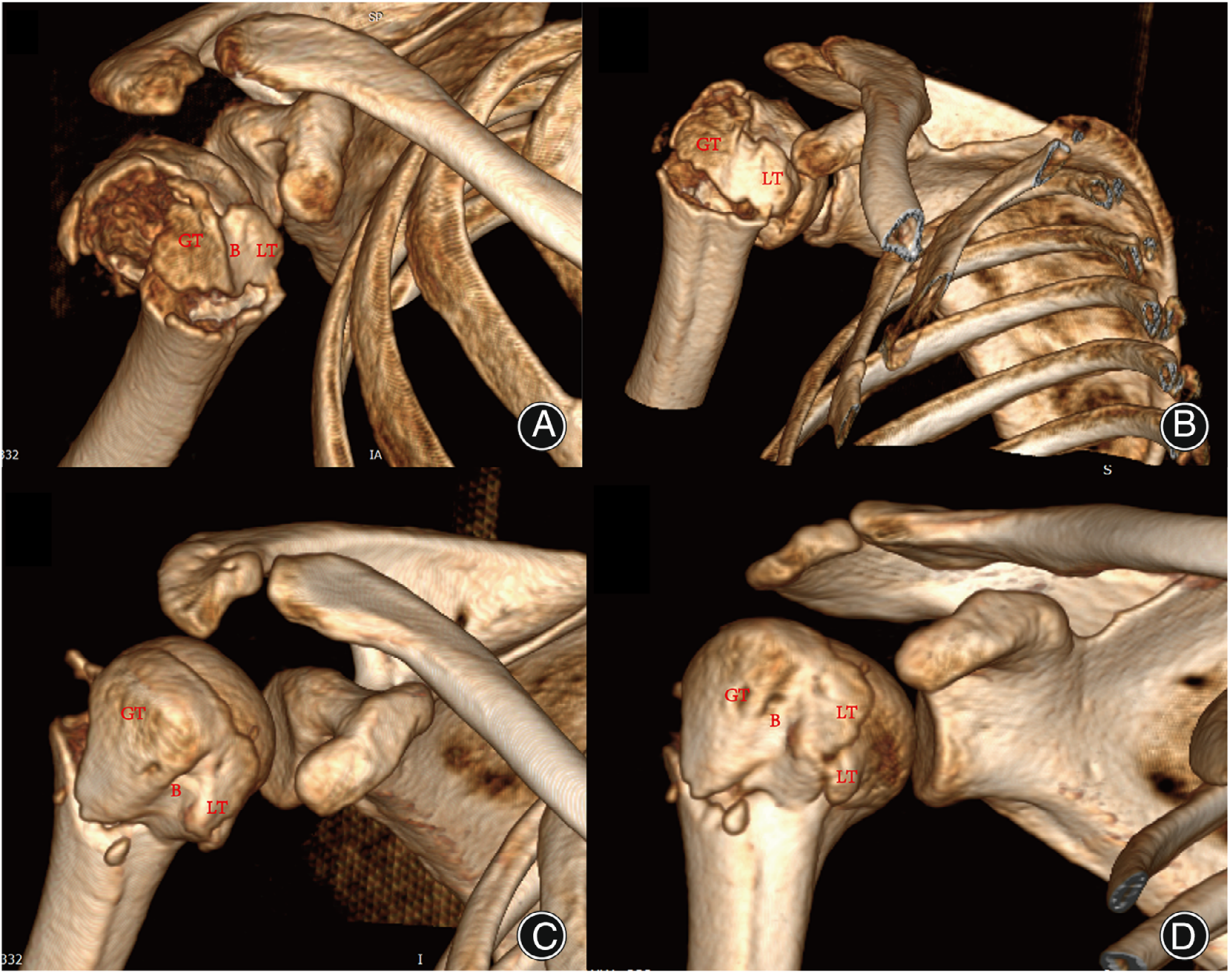
Shield fracture that presented with different patterns. (A) Fracture patient illustrated with a small segment of the greater tuberosity to the whole lesser tuberosity (B) The rotated 3D reconstruction illustrated the fracture pattern clearly with same patients in (A). (C) A fracture patient illustrated with a big segment of the greater tuberosity to the part of the lesser tuberosity. (D) The rotated 3D reconstruction illustrated the fracture pattern clearly with the same patients in (C). GT, greater tuberosity; LT, lesser tuberosity; B, bicipital groove

**Fig. 5:**
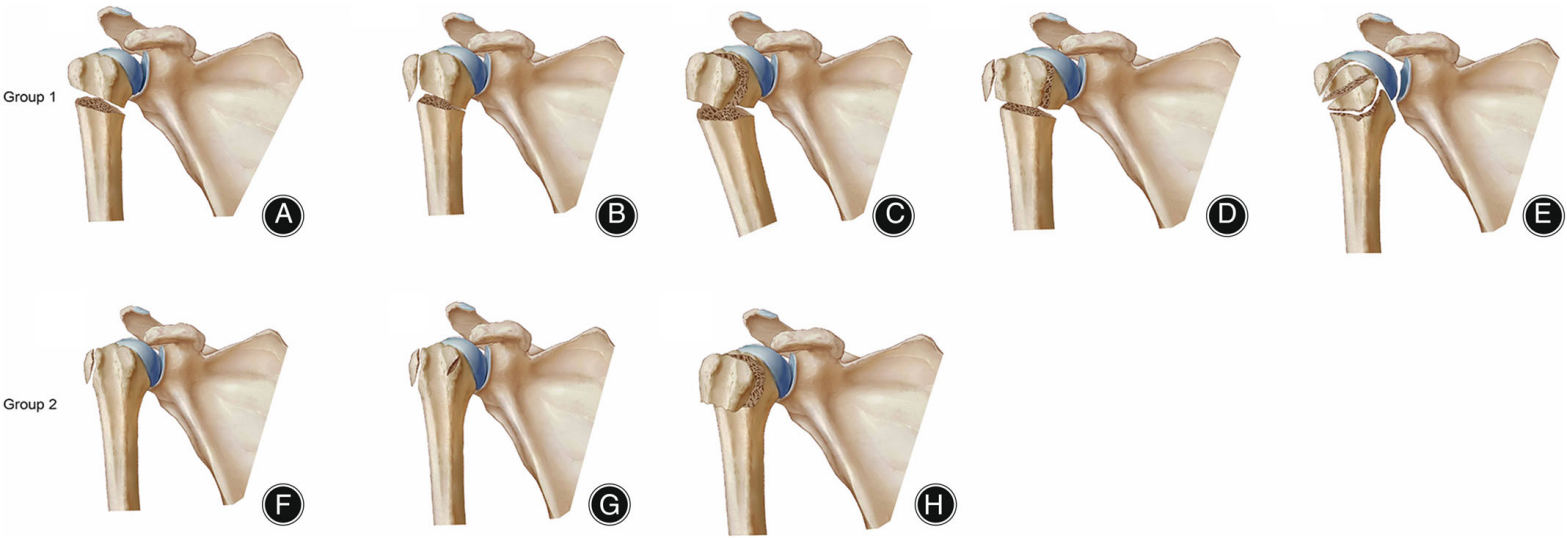
The presentation of different fractures in two groups. (A) Edelson I (surgical neck fracture); (B) Edelson II (surgical neck fracture and LT fracture); (C) Edelson III (Shield fracture); (D) Edelson IV (Shield variant fracture); (E) Bicipital groove fracture; (F) Edelson V (LT fracture); (G) Double tuberosity fracture; (H) Shell fracture

**Fig. 6:**
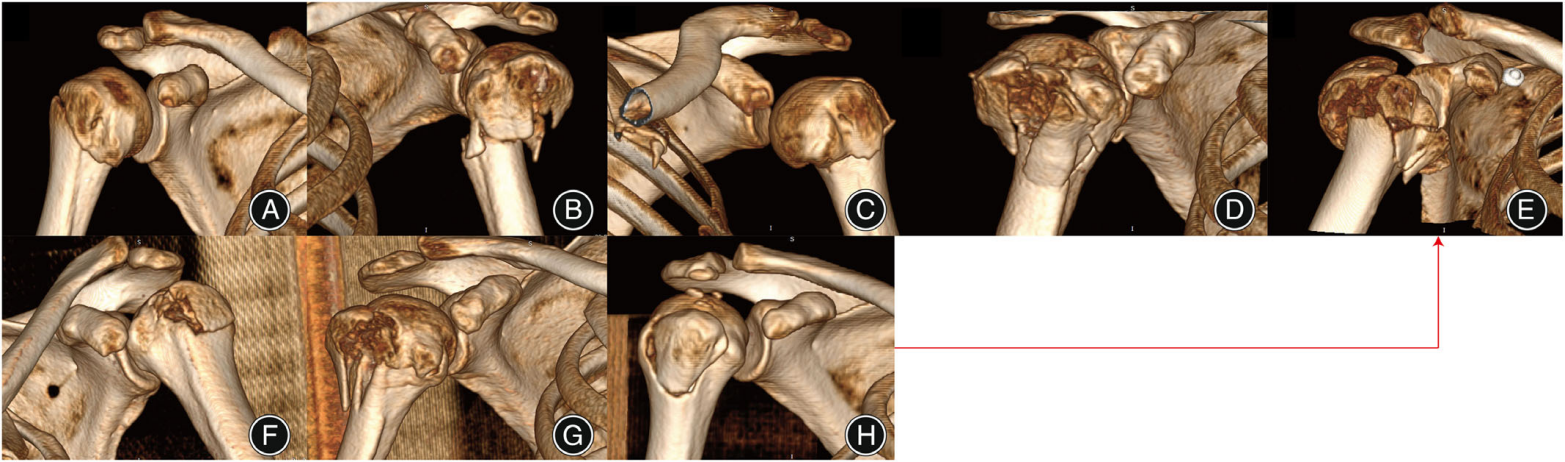
Examples of different fractures. Bicipital groove fracture was the final pattern of proximal humeral fracture in two groups. (A) Edelson I (surgical neck fracture); (B) Edelson II (surgical neck fracture and LT fracture); (C) Edelson III (Shield fracture); (D) Edelson IV (Shield variant fracture); (E) Bicipital groove fracture; (F) Edelson V (LT fracture); (G) Double tuberosity fracture; (H) Shell fracture

The bicipital groove was reported to be the strongest region in the proximal humerus.^9^ This area is a constraining closed space tunnel on which the transverse humeral ligament overlies.^11^ Furthermore, there is an important blood vessel named the anterior circumflex that supplies to the head in the region of the proximal bicipital groove. It is always used as an anatomical landmark to restore humeral head retroversion when conducting proximal humeral fracture arthroplasty. Theopold *et al*. also utilized the bicipital groove as an anatomic landmark and re‐established the shoulder function with a plate inserted in the groove after complex proximal humerus fractures.^12^ Different from previous research, there are many patients diagnosed as bicipital groove fracture in our research. The morphology and reduction of the bicipital groove fracture was a potential factor affecting the stability of the biceps tendon and functional outcomes of proximal fracture. Taylor conducted anatomical and histological research on bicipital tunnels and separated them into three zones. The distal margin of the subscapularis tendon (Zones 1 and 2) and proximal margin of the pectoralis major tendon (Zones 2 and 3) were anatomical landmarks for these three zones.^12^ The lesser tuberosity was the ending point of the subscapularis tendon, so the bicipital groove fracture above the distal end of the lesser tuberosity was Zone I (traditional bony bicipital groove). The bicipital groove fracture in our research was identified as the fracture located in Zone I. There were many proximal (Zone I) groove bicipital fractures, and most of the groove fracture injury (101) occurred along with surgical neck fracture, combine with greater or lesser tuberosity. Therefore, it can be concluded that the bicipital groove fracture was a common fracture type, and the main injury mechanism was high energy traumatic accident (Figs 4, 5, 6).

### Testify the Rationality of the Newly Revised Edelson Classifications based on Injury Mechanism of Each Main Type of Fracture

**Fig. 7:**
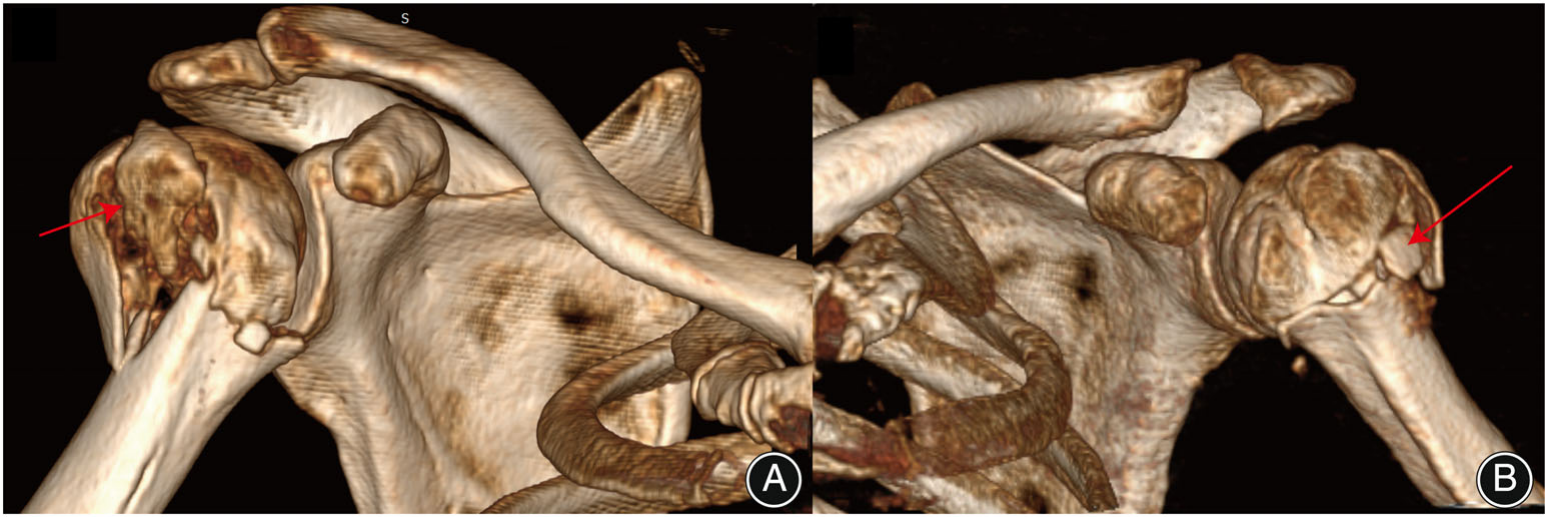
Fracture morphology with valgus or neutral position. (A) Valgus fracture classified as Edelson II illustrated a superior displaced or dissociative fracture fragment located upon the lateral part of the surgical neck fracture line. (B) A neutral fracture illustrated a fracture fragment located upon the lateral part of the surgical neck fracture

**Fig. 8:**
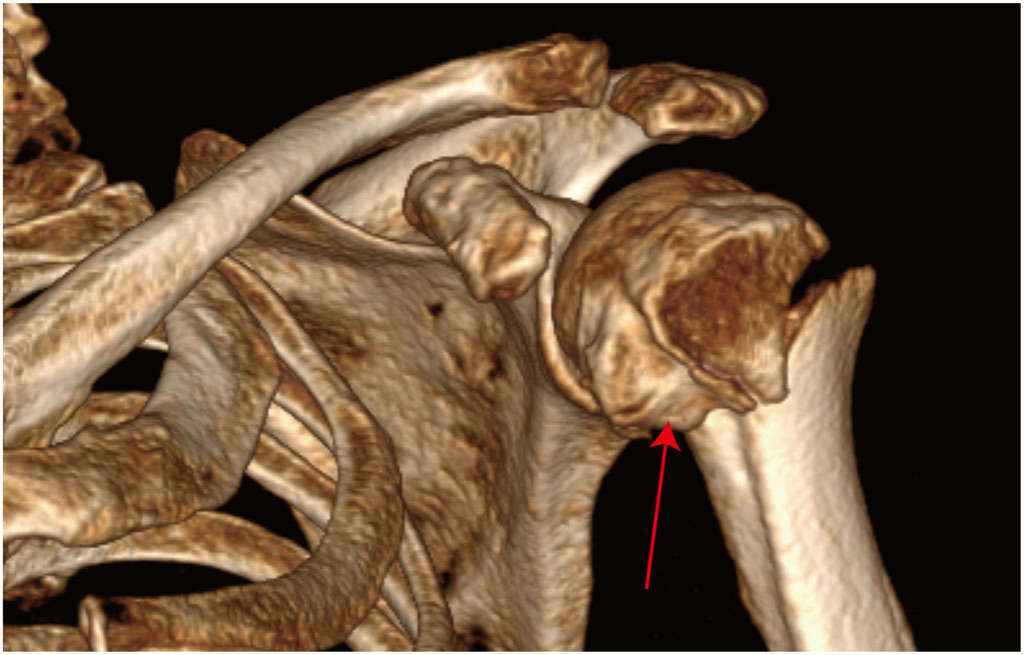
Fracture morphology with varus position. A varus fracture illustrated a superior displaced or partly overturned fracture fragment located upon the medial part of the surgical neck fracture line

Despite PHF is commonly observed in clinic, little is understood about the main underlying injury mechanism and fracture morphology which is essential to propose a new fracture classification. Currently, the most commonly observed injury mechanism of PHF is a direct impact onto the adducted shoulder in neutral rotation (side impact simulation) and a fall onto an outstretched hand (named as parachute reflex, means the shoulder is in a position of forward flexion, abduction, and internal rotation).^9^, ^13^, ^14^ To justify the new revised Edelson classification, a comprehensive analysis of injury mechanism and fracture morphology in each subtype was conducted. For traditional Edelson I, II, III, and IV and bicipital groove fractures, the main injury mechanism was the parachute reflex. Among them, Valgus and neutral positions were mostly observed. Edelson III should not be categorized as one individual classification due to its small number of fractures, and should be identified as a transitional fracture that belongs to Edelson IV. Therefore, the shoulder being in a position of forward flexion, abduction, and internal rotation (parachute reflex) was the main posture when the accident occurred in those fractures. The “parachute reflex” was then can be recognized as the main injury mechanism of those fractures, and the strong glenoid cavity bone of the proximal humeral joint acts as an anvil. With the upward force on distal humerus originated from force conduction, and the force originated from the upper part of the glenoid which counteract the upward force conducting along the humeral shaft, two forces were then dissipated through weaker areas of thin cortical bone. In simple terms, when the arm was internally rotated with an abducted position, the humeral head was supported by the glenoid. The humeral was then broken at the relatively weaker point (PHF). This also explains why most fractures in these fractures were found with abducted and neutral positions. It should be noted that patients with a neutral position were also classified into this mechanism because the fracture morphology was similar to the valgus morphology, which was characterized with the existence of dissociative or half‐free fragments surrounding the greater tuberosity (Fig.7). On the contrary, the varus fractures were characterized by depression fractures located surrounding the lesser tuberosity, and only 64 fractures were identified as varus fractures (Fig. 8).

For the isolated GT fracture, the main mechanism was impingement of the greater tuberosity against the acromion (verified by decompression on GT). Avulsion and split GT fractures were observed in 59 and 64 shoulders, respectively. However, the type of GT does not truly reflect the mechanism of injury, the existence of potential concomitant fracture was also act as a prompt. For example, it was noticed that many GT fractures were found with depressed fragments on the superior of the GT, especially in none or inferior displacement fractures. Nine fractures with superior displacement were also found with obvious depressed fractures. The depressed fracture always means a direct contact between GT and other most adjacent bone structure. Therefore, it was concluded that in isolated greater tuberosity fractures, especially with no or inferior displacement, impingement of the greater tuberosity against the acromion was the main injury mechanism. Mutch *et al*. divided GT fractures into three types: avulsion, split, and depression,^15^ but careful examination especially to the 3D reconstruction was needed to identify the true mechanism of proximal humeral injuries. For the dislocated GT fracture, shearing against the inferior glenoid rim was the main injury mechanism. The incidence of shoulder dislocation with GT fracture was 17.5% in our research. According to the results, in shoulder dislocations along with GT fractures patients, it appears that split but not avulsion was the leading mechanism of injury. Furthermore, no displacement or inferior displacement of the greater tuberosity was detected and accounted for 93.5% (29) of cases. One was displaced with a position of supination, and one was displaced with a position of pronation. Therefore, shearing against the glenoid rim seems to be a more convincing mechanism as a cause of greater tuberosity fractures.

Along those mentioned injury mechanism analysis, the relative connecting fracture types between five main types were also found such as double tuberosity fracture, shell fracture, and shield fracture. Finally, these two groups connected with each other tightly and ultimately result in bicipital fractures, which represent the most serious type of proximal humeral fracture caused by serious violent trauma. The injury mechanism and morphology of proximal humeral fracture became explicit in each group, and the connection between each fracture type was more closed. Based on above analysis, the new revised Edelson classification was proposed and it was helpful to guide the surgeon to establish the fracture reduction strategy efficiently compared with the traditional Edelson classifications.

### Limitations

The strength of the research was that injury mechanisms for different fracture types are rationalized along with a larger sample size, so it was more convincing to propose a new classification. Weaknesses of the study include the retrospective design; however, the injury mechanism and other information were reliable due to collection from the initial documentation. The main mechanism was discussed, but for the infrequently occurring fractures, their injury mechanism was not analyzed due to their relatively small numbers. However, the revised classification was still helpful to guide the treatment of proximal humeral fractures. It is believed that the revised classification has its own merits based on the injury mechanism and fracture morphology, and following up to those patients such as rehabilitation function, revision rate, complication and the new guidelines about fractures treatment strategy would be proposed in our further research.

**Conclusions**In conclusion, the bicipital groove fracture which has been ignored in previous research is proved to be a commonly observed fracture pattern, and included in the revised Edelson classification. The revised Edelson classification proposed based on injury mechanism was verified to be a good proximal humeral fracture classification with good reliability compared with traditional Edelson classification.

## Author Contributions

Conceptualization: Yingze Zhang; data curation: Sifan Yang, Xiaojuan Zhang; formal analysis: Weichong Dong; investigation: Yali Zhou, Meishuang Shang; methodology: Jialiang Guo; project administration: Jialiang Guo; resources: Yingze Zhang, Weichong Dong; software: Jialiang Guo; supervision: Zhiyong Hou, Yingze Zhang; validation: Zhiyong Hou, Yingze Zhang; visualization: Zhiyong Hou; writing – original draft: Jialiang Guo; and writing – review & editing: Jialiang Guo.

## Funding Information

The research was supported by National Natural Science Foundation of China (82002281) and Natural Science Foundation of Hebei (H2021206054), the Main Medical Scientific Research of Hebei (Award Number 20210543, 20221209), and China Postdoctoral Fund (2021 M701785). The funders had no role in study design, data collection and analysis, decision to publish, or preparation of the manuscript.

## References

[1] Jo YH , Lee KH , Lee BG . Surgical trends in elderly patients with proximal humeral fractures in South Korea: a population‐based study. BMC Musculoskelet Disord. 2019;20(1):136. 30927910 10.1186/s12891-019-2515-2 PMC6441205

[2] Du S , Ye J , Chen H , Li X , Lin Q . Interventions for treating 3‐ or 4‐part proximal humeral fractures in elderly patient: a network meta‐analysis of randomized controlled trials. Int J Surg. 2017;48:240–6. 28890408 10.1016/j.ijsu.2017.09.002

[3] Myeroff CM , Anderson JP , Sveom DS , Switzer JA . Predictors of mortality in elder patients with proximal humeral fracture. Geriatr Orthop Surg Rehabil. 2018;9:2151458517728155. 29560284 10.1177/2151458517728155 PMC5851103

[4] Strohm PC , Helwig P , Konrad G , Südkamp NP . Locking plates in proximal humerus fractures. Acta Chir Orthop Traumatol Cech. 2007;74(6):410–5. 18198093

[5] Sumrein BO , Mattila VM , Lepola V , Laitinen MK , Launonen AP , NITEP Group . Intraobserver and interobserver reliability of recategorized Neer classification in differentiating 2‐part surgical neck fractures from multi‐fragmented proximal humeral fractures in 116 patients. J Shoulder Elbow Surg. 2018;27(10):1756–61. 29866397 10.1016/j.jse.2018.03.024

[6] Ferrel JR , Trinh TQ , Fischer RA . Reverse total shoulder arthroplasty versus hemiarthroplasty for proximal humeral fractures: a systematic review. J Orthop Trauma. 2015;29(1):60–8. 25186842 10.1097/BOT.0000000000000224

[7] Yahuaca BI , Simon P , Christmas KN , Patel S , Gorman RA 2nd , Mighell MA , et al. Acute surgical management of proximal humerus fractures: ORIF vs. hemiarthroplasty vs. reverse shoulder arthroplasty. J Shoulder Elbow Surg. 2020;29(7s):32–40. 10.1016/j.jse.2019.10.012 31948835

[8] Brorson S , Olsen BS , Frich LH , Jensen SL , Sørensen AK , Krogsgaard M , et al. Surgeons agree more on treatment recommendations than on classification of proximal humeral fractures. BMC Musculoskelet Disord. 2012;13:114. 22738149 10.1186/1471-2474-13-114 PMC3495208

[9] Edelson G , Kelly I , Vigder F , Reis ND . A three‐dimensional classification for fractures of the proximal humerus. J Bone Joint Surg Br. 2004;86(3):413–25. 15125131 10.1302/0301-620x.86b3.14428

[10] Capriccioso CE , Zuckerman JD , Egol KA . Initial varus displacement of proximal humerus fractures results in similar function but higher complication rates. Injury. 2016;47(4):909–13. 26878816 10.1016/j.injury.2016.01.021

[11] Taylor SA , Fabricant PD , Bansal M , Khair MM , McLawhorn A , DiCarlo EF , et al. The anatomy and histology of the bicipital tunnel of the shoulder. J Shoulder Elbow Surg. 2015;24(4):511–9. 25457787 10.1016/j.jse.2014.09.026

[12] Theopold J , Marquaß B , Fakler J , Steinke H , Josten C , Hepp P . The bicipital groove as a landmark for reconstruction of complex proximal humeral fractures with hybrid double plate osteosynthesis. BMC Surg. 2016;16:10. 26968940 10.1186/s12893-016-0125-6 PMC4788826

[13] Majed A , Thangarajah T , Southgate DF , Reilly P , Bull A , Emery R . The biomechanics of proximal humeral fractures: injury mechanism and cortical morphology. Shoulder Elbow. 2019;11(4):247–55. 31316585 10.1177/1758573218768535 PMC6620795

[14] Charlton IW , Johnson GR . A model for the prediction of the forces at the glenohumeral joint. Proc Inst Mech Eng H. 2006;220(8):801–12. 17236514 10.1243/09544119JEIM147

[15] Mutch J , Laflamme GY , Hagemeister N , Cikes A , Rouleau DM . A new morphological classification for greater tuberosity fractures of the proximal humerus: validation and clinical implications. Bone Joint J. 2014;96(5):646–51. 24788500 10.1302/0301-620X.96B5.32362

